# Characterisation and outcomes of ARDS secondary to pneumonia in patients with and without SARS-CoV-2: a single-centre experience

**DOI:** 10.1136/bmjresp-2020-000731

**Published:** 2020-11-30

**Authors:** Rahul Y Mahida, Minesh Chotalia, Joseph Alderman, Chhaya Patel, Amber Hayden, Ruchi Desai, Emily Beesley, Louise E Crowley, Marina Soltan, Mansoor Bangash, Dhruv Parekh, Jaimin Patel, David R Thickett

**Affiliations:** 1 Birmingham Acute Care Research Group, Institute of Inflammation and Ageing, University of Birmingham, Birmingham, UK; 2 Department of Anaesthesia and Critical Care, University Hospitals Birmingham NHS Foundation Trust, Birmingham, Birmingham, UK; 3 School of Medical Sciences, The University of Manchester, Manchester, Manchester, UK; 4 School of Medical and Dental Sciences, University of Birmingham, Birmingham, Birmingham, UK

**Keywords:** ARDS, viral infection, pneumonia

## Abstract

**Introduction:**

Acute respiratory distress syndrome (ARDS) is the major cause of mortality in patients with SARS-CoV-2 pneumonia. It appears that development of ‘cytokine storm’ in patients with SARS-CoV-2 pneumonia precipitates progression to ARDS. However, severity scores on admission do not predict severity or mortality in patients with SARS-CoV-2 pneumonia. Our objective was to determine whether patients with SARS-CoV-2 ARDS are clinically distinct, therefore requiring alternative management strategies, compared with other patients with ARDS. We report a single-centre retrospective study comparing the characteristics and outcomes of patients with ARDS with and without SARS-CoV-2.

**Methods:**

Two intensive care unit (ICU) cohorts of patients at the Queen Elizabeth Hospital Birmingham were analysed: SARS-CoV-2 patients admitted between 11 March and 21 April 2020 and all patients with community-acquired pneumonia (CAP) from bacterial or viral infection who developed ARDS between 1 January 2017 and 1 November 2019. All data were routinely collected on the hospital’s electronic patient records.

**Results:**

A greater proportion of SARS-CoV-2 patients were from an Asian ethnic group (p=0.002). SARS-CoV-2 patients had lower circulating leucocytes, neutrophils and monocytes (p<0.0001), but higher CRP (p=0.016) on ICU admission. SARS-CoV-2 patients required a longer duration of mechanical ventilation (p=0.01), but had lower vasopressor requirements (p=0.016).

**Discussion:**

The clinical syndromes and respiratory mechanics of SARS-CoV-2 and CAP-ARDS are broadly similar. However, SARS-CoV-2 patients initially have a lower requirement for vasopressor support, fewer circulating leukocytes and require prolonged ventilation support. Further studies are required to determine whether the dysregulated inflammation observed in SARS-CoV-2 ARDS may contribute to the increased duration of respiratory failure.

**Key messages**

- This is the first UK study comparing the clinical characteristics and outcomes of acute respiratory distress syndrome (ARDS) secondary to pneumonia in patients with and without SARS-CoV-2.
- Are patients with SARS-CoV-2 ARDS clinically distinct to other patients with ARDS, therefore, requiring alternative management strategies?
- While the clinical syndromes of ARDS secondary to SARS-CoV-2 and community-acquired pneumonia are similar, SARS-CoV-2 patients initially have a lower requirement for vasopressor support and require prolonged ventilation support.

## Introduction

SARS-CoV-2 pneumonia can progress to hypoxaemic respiratory failure requiring mechanical ventilation, with patients fulfilling the Berlin criteria for acute respiratory distress syndrome (ARDS).^1–3^ Intensive care unit (ICU) mortality rates of up to 68% from SARS-CoV-2 ARDS have been reported.^3 4^ A multinational study undertaken prior to the SARS-CoV-2 pandemic found that pneumonia was the underlying risk factor in 59% of ARDS cases.^5^ Recently, a retrospective cohort study undertaken in Wuhan, China found that 41.8% of adult patients admitted with SARS-CoV-2 pneumonia developed ARDS.^6^ Risk factors for mortality from SARS-CoV-2 pneumonia include increasing age, coronary heart disease, diabetes mellitus and chronic kidney disease.^7 8^ Admission CURB-65 scores do not predict severity or mortality in patients with SARS-CoV-2 pneumonia, due to a rapidly progressing clinical course.^7^ However, lymphopenia, eosinopenia and elevated acute phase proteins are predictors of increased disease severity.^7 9 10^

It appears that development of ‘cytokine storm’ in patients with SARS-CoV-2 pneumonia is associated with progression to ARDS, however, the cytopathic effects of the viral pneumonia may be just as important.^9 11 12^ Histological analysis of postmortem lung tissue from SARS-CoV-2 pneumonia patients has shown diffuse alveolar damage (DAD).^13 14^ The presence of DAD has previously been used to identify a subphenotype of ARDS with higher mortality.^15 16^ These findings suggest that a similar pathological process occurs in patients with ARDS with and without SARS-CoV-2.

Our objective was to determine whether patients with SARS-CoV-2 ARDS are clinically distinct, therefore, requiring alternative management strategies, compared with other patients with ARDS.^17^ This retrospective study provides clinical characterisation of ARDS patients with and without SARS-CoV-2 admitted to a single-centre ICU.

## Methods

This is a single-centre, observational, retrospective study from the ICU of the Queen Elizabeth Hospital Birmingham, UK. All data were routinely collected on the hospital’s electronic patient records. Only data that were obtained as part of routine clinical care were collected for this study. All data were anonymised and entered by the Local Clinical Care Team, without linkage to any patient identifiers, in accordance with national and local guidance.

Two ICU cohorts of patients were analysed: SARS-CoV-2 pneumonia patients admitted between 11 March and 21 April 2020 (online supplemental figure 1) and all patients with community-acquired pneumonia (CAP) from bacterial or viral infection who developed ARDS between 1 January 2017 and 1 November 2019 (online supplemental figure 2). Patients who developed hospital-acquired pneumonia (HAP: defined as onset >48 hours after hospital admission) were excluded. This was to identify a more relevant, directly comparable control group, in which infection was acquired in the community, and pneumonia was present at hospital admission. The causative organisms and clinical course of CAP and HAP also differ significantly. Patients with ARDS secondary to others causes were also excluded. The sample sizes were determined pragmatically, to include all SARS-CoV-2 pneumonia patients admitted to the ICU within the first 6 weeks of the pandemic. The sample size of the CAP-ARDS control group included all such patients within the 3 years preceding the pandemic, since all these patients would have their hospital records within a rapidly accessible electronic system, and would have received protocolised management similar to that received by the SARS-CoV-2 ARDS patients.

10.1136/bmjresp-2020-000731.supp1 Supplementary data

As patients were from the same institution, their management prior to ICU admission and on ICU were broadly similar following local evidence-based protocols and national guidelines^5^ with respect to interventions that affect outcome including low tidal volume ventilation and prone positioning (online supplemental table 1). High frequency oscillatory ventilation was only used as a rescue therapy in patients with refractory severe respiratory failure who were not accepted by an extracorporeal membrane oxygenation centre, and were managed as per previously published algorithms.^18^ All patients were intubated, sedated and mechanically ventilated with positive pressure ventilation. Baseline demographic, comorbidities, laboratory investigations, physiological parameters and severity scores (Acute Physiology And Chronic Health Evaluation II [APACHE II], Sequential Organ Failure Assessment [SOFA] and Murray Lung Injury) were collected at ICU admission. Sequential physiological and laboratory parameters were collected for 7 days whist on ICU. Sequential data are not available for all patients due to deaths of 15 SARS-CoV-2 and 5 CAP-ARDS patients within 1 week.

Statistical analysis was performed using GraphPad Prism version 8.0. Data distributions were non-parametric and are presented as median with IQR for continuous variables and number (percentage) for categorical variables. Differences between patient groups were analysed using Mann-Whitney-U test for continuous data and Fisher’s exact test for categorical data. Two-sided tests were used for all comparisons with p<0.05 considered statistically significant.

## Results

A total of 111 patients with SARS-CoV-2 ARDS and 29 patients with CAP-ARDS met the inclusion criteria (table 1). Many patients (n=33) screened for CAP-ARDS were excluded, as pneumonia had developed >48 hours after hospital admission. Patient demographic details are shown in table 1, with both groups being broadly similar except for ethnic background. A greater proportion of SARS-CoV-2 patients were of Asian/Asian British ethnicity (p=0.002), and a lower proportion were of White ethnicity (p=0.012), compared with CAP-ARDS patients

**Table 1:** Demographics, laboratory and physiological characteristics of SARS-CoV-2 ARDS and CAP-ARDS patients on admission to ICU

	SARS-CoV-2 ARDS (n=111)	CAP-ARDS (n=29)	P value
**Demographics**			
Age at admission (years)	56 (47–63)	55 (41–59)	0.315*
Gender, male (n, %)	84 (75.7%)	19 (65.5%)	0.358†
Body mass index	29 (27–34)	29 (26–33)	0.403*
Ethnicity			
White	54 (48.6%)	22 (75.8%)	**0.012†**
Asian/Asian British	34 (30.6%)	1 (3.4%)	**0.002†**
Black/African/Caribbean	9 (8.1%)	0 (0%)	0.204†
Mixed/multiple	3 (2.7%)	1 (3.4%)	0.999†
Other	10 (9.0%)	5 (17.2%)	0.308†
Comorbidities			
None	30 (27.0%)	10 (34.5%)	0.490†
Hypertension	44 (39.6%)	9 (31.0%)	0.520†
Obesity	55 (49.5%)	12 (41.4%)	0.532†
Ischaemic heart disease	6 (5.4%)	1 (3.4%)	>0.999†
Diabetes	33 (29.7%)	4 (13.8%)	0.100†
Asthma/COPD	12 (10.8%)	4 (13.8%)	0.743†
Stroke/TIA	3 (2.7%)	1 (3.4%)	0.999†
Chronic kidney disease	9 (8.1%)	1 (3.4%)	0.688†
Cancer	7 (6.3%)	3 (10.3%)	0.432†
**Severity scoring**			
APACHE II	14 (12–18)	18 (16–24)	0.0002*
SOFA Score	8 (7–10)	12 (9–14)	<0.0001*
Murray Lung Injury Score	2.75 (2.5–3.0)	2.75 (2.33–3.00)	0.645*
**Laboratory parameters on ICU admission**			
White cell count (x10^9^/L)	9.0 (5.9–12.6)	14.6 (10.6–22.9)	<0.0001*
Neutrophils (x10^9^/L)	6.9 (4.5–10.2)	12.7 (9.0–21.0)	<0.0001*
Lymphocyte (x10^9^/L)	0.88 (0.57–1.20)	0.7 (0.5–1.2)	0.327*
Monocytes (x10^9^/L)	0.43 (0.29–0.65)	0.9 (0.6–1.3)	<0.0001*
Eosinophils (x10^9^/L)	0 (0–0.03)	0 (0–0.1)	0.277*
CRP (mg/L)	172 (113–241)	91 (40–235)	0.016*
Platelets (10^9^/L)	224 (174–305)	191 (111–294)	0.029*
Creatinine (µmol/L)	77 (64–111)	87 (67–178)	0.260*
Bilirubin (µmol/L)	12 (9–20)	18 (8–45)	0.141*
Albumin (g/L)	27 (24–32)	31 (27–35)	0.003*
**Ventilator parameters on ICU admission**			
Ppeak (cmH_2_O)	27 (24–30)	28 (24–30)	0.578*
PEEP (cmH_2_O)	10 (8–12)	8 (6–10)	0.003*
Driving pressure (cmH_2_O)	16 (14–19)	19 (14–22)	0.178*
Tidal volume (mL/ kg)	5.11 (4.60–5.89)	5.98 (4.87–6.96)	0.013*
Pulmonary compliance (mL/cmH_2_O)	28 (24–34)	25 (22–34)	0.471*
FiO_2_ (%)	70 (60–86)	80 (60–100)	0.149*
**ICU outcomes**			
Hospital mortality	40 (36.0%)	12 (41.4%)	0.668†
Time to death from ICU admission (days)	11 (8–18)	11 (7–17)	0.874*
ICU LoS (days)	17 (10–24)	13 (9–24)	0.344*
ARDS (PaO_2_ / FiO_2_ ratio kPa)			
Mild (>26.6–40)	6 (5.4%)	2 (6.9%)	0.670†
Moderate (>13.3 ≤26.6)	58 (52.3%)	15 (51.7%)	0.999†
Severe (≤13.3)	47 (42.3%)	12 (41.4%)	0.999†
Day 1 PaO_2_ / FiO_2_ ratio (kPa)	15 (12–17)	15 (11–17)	0.895*
Duration of mechanical ventilation (days)	15 (9–20)	9 (3–17)	0.012*
Maximum norepinephrine dose on day 1 of ICU admission (**µ**g/kg/min)	0.067 (0.015–0.120)	0.490 (0–0.623)	0.016*
Need for RRT	46 (41.4%)	11 (37.9%)	0.833†
Need for tracheostomy	55 (49.5%)	16 (55.2%)	0.678†

Data are n (%) or median (IQR). Tidal volume calculated using predicted body weight.

*Represents p-values from a Mann-Whitney U test.

†Represents p-values from a Fisher’s exact test.

APACHE II, Acute Physiology And Chronic Health Evaluation II; ARDS, acute respiratory distress syndrome; CAP, community-acquired pneumonia; COPD, chronic obstructive pulmonary disease; FiO_2_, fraction of inspired oxygen; ICU, intensive care unit; LoS, length of stay; PaO_2_, partial pressure of oxygen; PEEP, positive end-expiratory pressure; RRT, renal replacement therapy; SOFA, Sequential Organ Failure Assessment.

**Figure 1:**
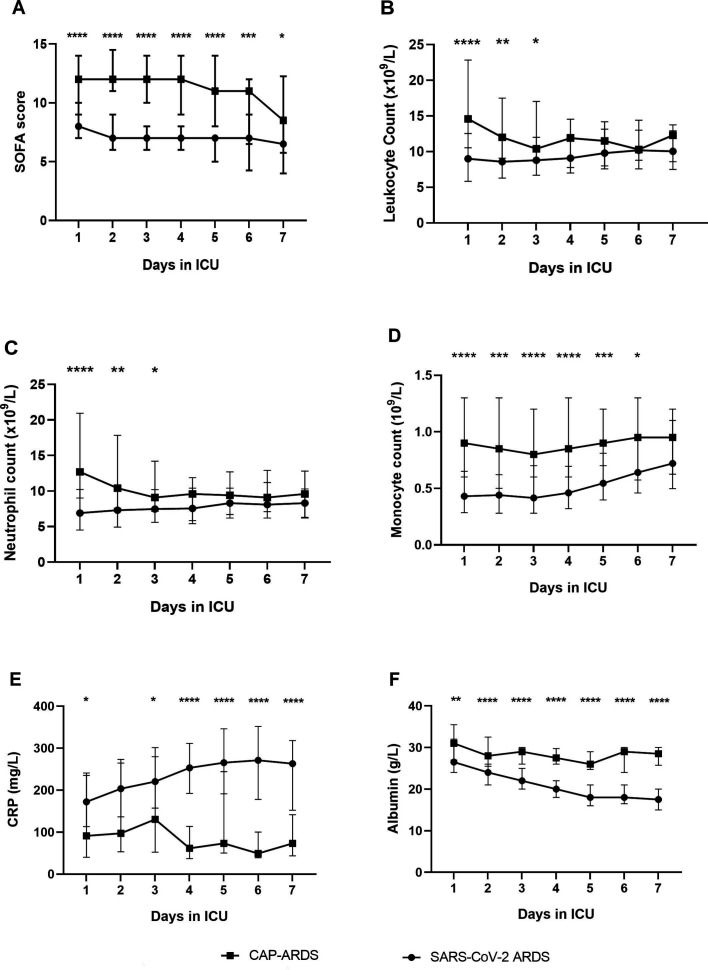
SOFA score and haematological parameters for SARS-CoV-2 and CAP-ARDS patients over the first 7 days in the ICU. (A) SOFA score. B: leucocyte count. (C): neutrophil count. (D) monocyte count. (E): CRP. (F): albumin. Data presented as daily median values and IQRs for SARS-CoV-2 ARDS and CAP-ARDS patients. A Mann-Whitney U test was performed at each time point to compare both patient groups: *P<0.05, **p<0.01, ***p<0.001, ****p<0.0001. ARDS, acute respiratory distress syndrome; CAP, community-acquired pneumonia; ICU, intensive care unit; SOFA, Sequential Organ Failure Assessment Score.

On ICU admission, SARS-CoV-2 patients had significantly lower APACHE-II and SOFA scores than CAP-ARDS patients (see table 1: p<0.0001). SOFA scores remained lower in SARS-CoV-2 patients for 7 days following ICU admission (figure 1A).

SARS-CoV-2 patients had lower circulating leukocytes, neutrophils and monocytes (p<0.0001 for all) than CAP-ARDS patients on ICU admission. Leucocytes and neutrophil counts remained lower in SARS-CoV-2 patients for 3 days following ICU admission, whereas monocyte counts remained lower for 6 days (figure 1B–D). Albumin was lower (p=0.003) while CRP (p=0.016) and platelet count (p=0.029) were higher at ICU admission in SARS-CoV-2 patients. Differences in CRP and albumin between patient groups increased with duration of ICU stay (figure 1E, F). There was no difference in lymphocytes, eosinophils, bilirubin or creatinine between groups on ICU admission.

Positive end-expiratory pressure (PEEP) was higher (p=0.003) and tidal volumes were lower (p=0.006) in SARS-CoV-2 patients on ICU admission. However, there was no difference in other ventilator parameters between groups on ICU admission, including driving pressure, peak inspiratory pressure, pulmonary compliance, fraction of inspired oxygen and PaO_2_ / FiO_2_ ratio. SARS-CoV-2 patients required a lower dose of vasopressors on ICU admission (p=0.016).

SARS-CoV-2 patients required a longer duration of mechanical ventilation compared with CAP-ARDS patients (p=0.010). However, there was no significant difference in other major ICU outcomes between groups, including hospital mortality, ICU length of stay, time to death from ICU admission, development of moderate/severe ARDS, need for renal replacement therapy or need for tracheostomy.

## Discussion

The stark difference between patient numbers indicates that before the emergence of SARS-CoV-2, development of CAP-ARDS was comparatively rare. In keeping with previous findings, our study confirms that SARS-CoV-2 pneumonia seems to disproportionately affect patients from some ethnic minority backgrounds compared with CAP-ARDS.^19^

Patients with SARS-CoV-2 ARDS develop rapid respiratory failure, however other organ functions seem to be initially preserved, with reduced requirement for vasopressors on ICU admission. Severity scores (SOFA, APACHE-II) were higher in the CAP-ARDS group on ICU admission. Similar observations were made in a study comparing patients with SARS-CoV-2 versus H1N1 Influenza,^20^ suggesting that SARS-CoV-2 pneumonia initially causes less severe ARDS compared with CAP-ARDS patients who present with mainly bacterial infections or Influenza. However, the increased duration of respiratory failure in SARS-CoV-2 ARDS patients indicates that existing severity scores may not be predictive in this population.

The lower circulating leucocyte and neutrophil count in SARS-CoV-2 ARDS is similar to that observed in a previous paediatric study comparing pneumonia patients with SARS-CoV-2 vs Influenza A.^21^ However, in contrast to this study, we found that CRP was significantly elevated in SARS-CoV-2 patients. Reduced circulating leukocytes in SARS-CoV-2 ARDS patients may indicate a greater migration of neutrophils and monocytes into the alveolar space, impaired leukopoiesis or increased leucocyte clearance. Further studies are required to elucidate the relationship between the observed elevated acute phase proteins and lower circulating leucocytes, which may lead to a greater understanding of SARS-CoV-2 pathogenesis.

While the duration of mechanical ventilation in CAP-ARDS patients was similar to previous ARDS cohorts,^5^ the SARS-CoV-2 patients required an increased duration of mechanical ventilation. However, there was no significant difference in most ventilator parameters or other major ICU outcomes (eg, mortality and ICU length of stay) between patient groups. Absolute differences in PEEP and tidal volume between patient groups on ICU admission were small. The requirement for prolonged ventilation support is a key feature of SARS-CoV-2 ARDS, which otherwise causes a clinical syndrome similar to that observed in CAP-ARDS.

Although our CAP-ARDS tracheostomy rates may be considered high by some standards,^5^ they are similar to those in other primary ARDS cohorts from European nations,^22^ and thus they do not reflect outlying clinical behaviour. Our recent article currently in press (https://bjanaesthesia.org/article/S0007-0912(20)30678-4/fulltext) shows that we did not treat our SARS-CoV-2 patients any differently to our CAP-ARDS patients with regards to tracheostomy decisions, hence there being no significant difference between the groups.

A recent multicentre study by Grasselli *et al* has also compared SARS-CoV-2 ARDS patients with an earlier ARDS patient cohort unrelated to SARS-CoV-2.^23^ In contrast to our results, lung compliance was found to be reduced in SARS-CoV-2 ARDS patients. A subset of patients with SARS-CoV-2 ARDS with low compliance and elevated D-dimers were found to have an increased risk of mortality. Our study may have been underpowered to detect a difference in compliance, as the number of patients included within our study was lower. However, the control group used by Grasselli *et al* was significantly different to ours, with pneumonia being the underlying aetiology in only 57.6% of these patients, compared with 100% in our study.^23^

Several laboratory parameters relevant to SARS-CoV-2 patients including ferritin, lactate dehydrogenase and d-dimer^6 23^ were not included in our comparison, because only a small minority of patients in the CAP-ARDS control group had received these investigations. Data regarding the incidence of ARDS in CAP patients were also not available. Despite inclusion of all patients meeting the eligibility criteria at our centre, one of the largest ICU facilities in Europe, the power of our study was low due to the small size of the CAP-ARDS cohort. There is also a temporal bias in our data, with the SARS-CoV-2 cases being newer. These are significant limitations of our study. Another important limitation of our study is its single-centre observational nature, thus its applicability to a broader range of patients is difficult.

In summary, we show that while the respiratory mechanics of SARS-CoV-2 and CAP-ARDS patients are similar, SARS-CoV-2 patients initially have a lower requirement for vasopressor support, fewer circulating leucocytes and require prolonged ventilation support. We do not recommend changes to the current management of SARS-CoV-2 ARDS based on this study. However, further studies are required to determine whether the dysregulated inflammation observed in SARS-CoV-2 patients contributes to the increased duration of respiratory failure.
