# Supplementary material for: Characterisation and outcomes of ARDS secondary to pneumonia in patients with and without SARS-CoV-2: a single-centre experience
Source: BMJ Open Respir Res. 2020 Nov 30;7(1):e000731. doi: 10.1136/bmjresp-2020-000731 (PMC7705425; doi:10.1136/bmjresp-2020-000731)
Supplement: Supplementary data [file bmjresp-2020-000731supp001.pdf]

Supplemental Information

Figure S1: CONSORT flowsheet for SARS-CoV-2 ARDS patients

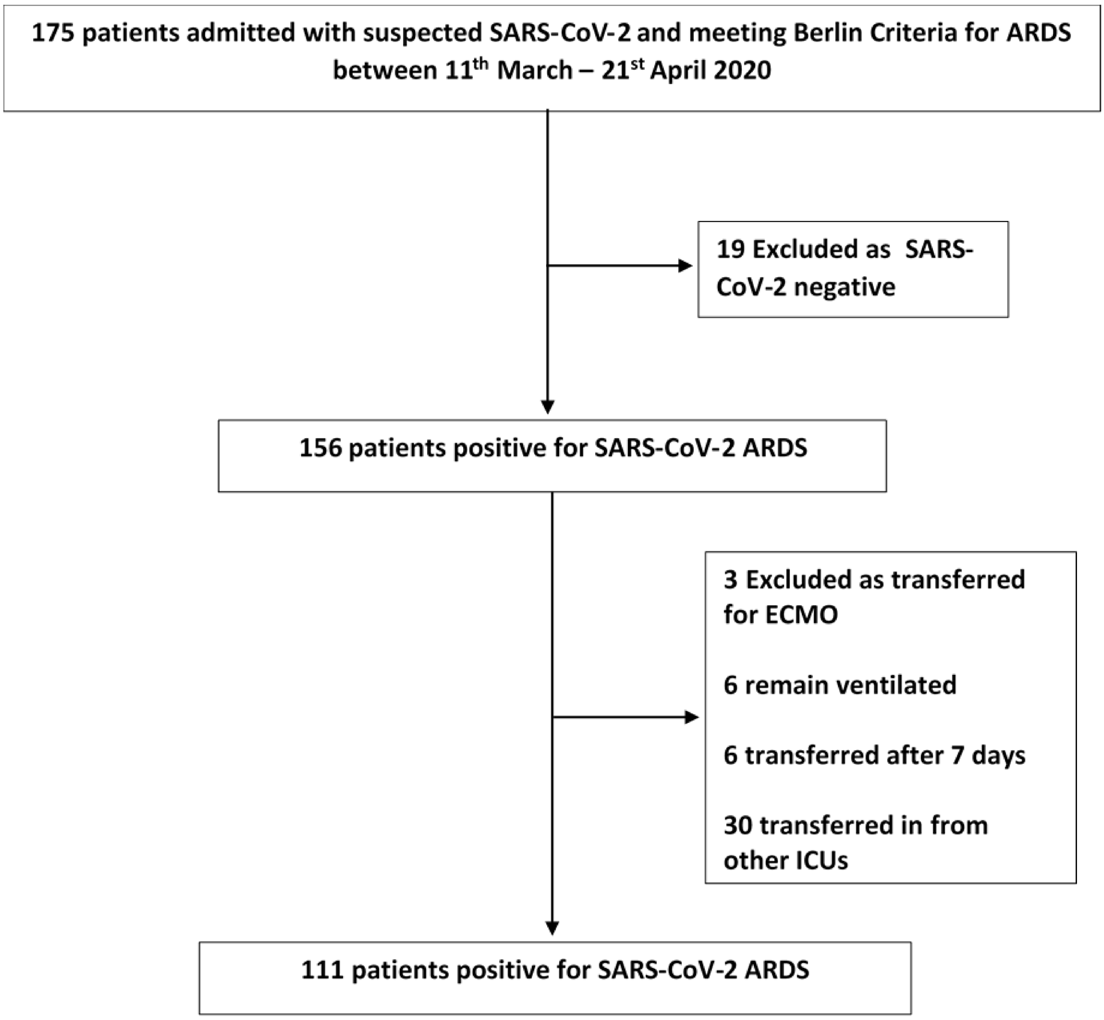

Abbreviations

ARDS: Acute Respiratory Distress Syndrome. ECMO: Extra-corporeal membrane oxygenation. ICU = Intensive Care Unit. SARS-CoV-2 = Severe Acute Respiratory Syndrome Coronavirus 2.

Figure S2: CONSORT flowsheet for CAP-ARDS patients

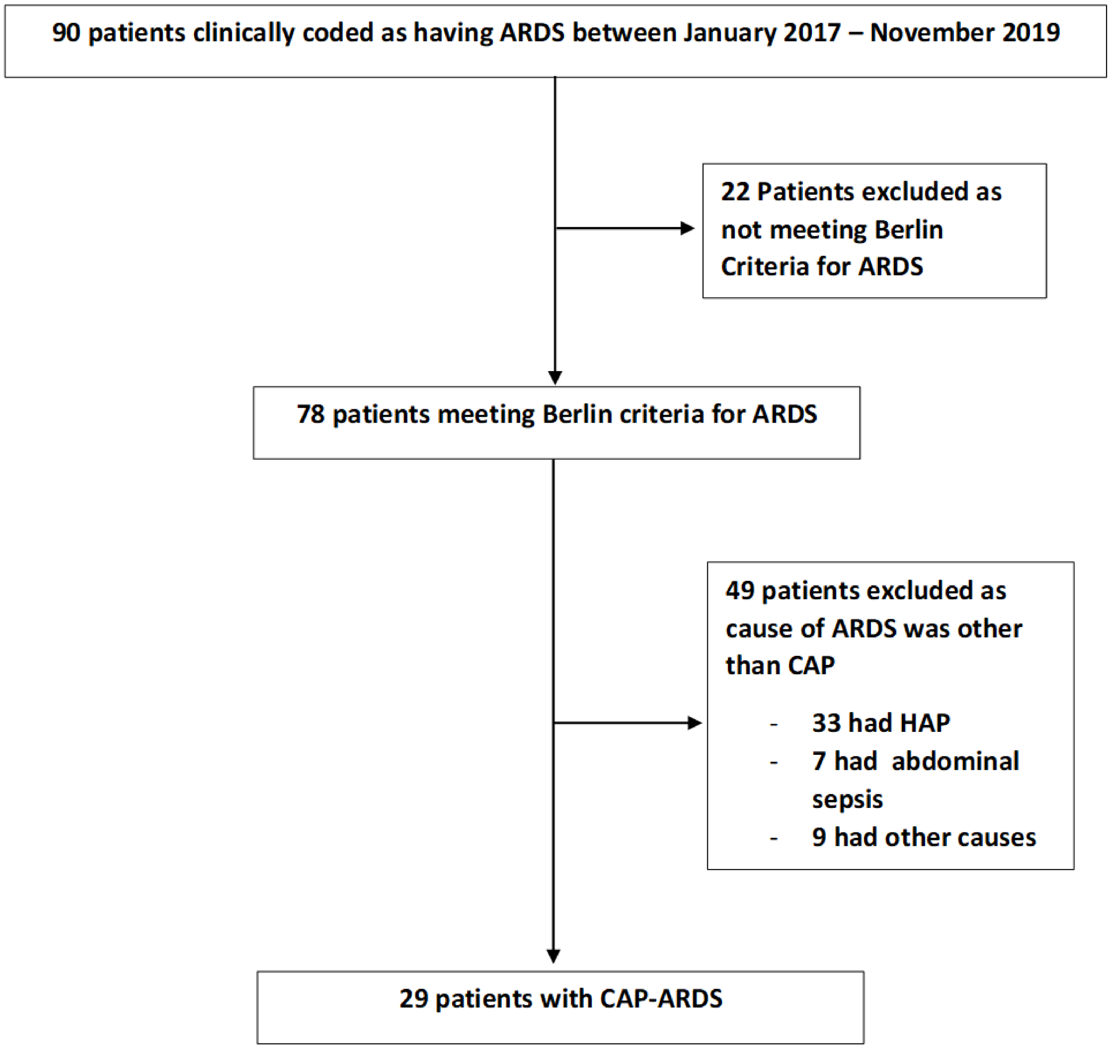

Abbreviations

ARDS: Acute Respiratory Distress Syndrome. CAP = Community-Acquired Pneumonia. HAP: Hospital-Acquired Pneumonia. ICU = Intensive Care Unit. SARS-CoV-2 = Severe Acute Respiratory Syndrome Coronavirus 2.

Table S1: Summary of the protocolised management instituted at our hospital

Ventilation	
Tidal Volume	6-8 ml/kg/PBW
Ppeak/Plateau	<30 cmH_2O
PaO_2	Target 8-10 kPa
$PaCO_2$	Target to keep pH>7.2
Prone Position	18 hours if FiO_2 ≥0.6
Neuromuscular Blockade	Atracurium infusion
Fluid Balance	Target for neutral balance. Enteral feeds for excluded in fluid balance
Antibiotics	Cessation of routine antibiotics for community acquired pneumonia if SARS-CoV-2 positive
Sedation	Morphine and Midazolam
First-line vasopressor	Noradrenaline
Thromboprophylaxis	<50kg Enoxaparin 40mg daily s/c<br>50 - 150kg Enoxaparin 40mg twice daily s/c<br>>150kg Enoxaparin 60mg twice daily s/c<br>GFR < 30 mls / min reduce dose by 50%

Abbreviations

FiO_2 = Fraction of inspired oxygen. GFR = Glomerular filtration rate. S/C = Subcutaneous. PaO_2/CO_2 = Partial pressure of oxygen / carbon dioxide. PWB = Predicted body weight.
